# Cross-cultural validity of a dietary questionnaire for studies of dental caries risk in Japanese

**DOI:** 10.1186/1472-6831-14-1

**Published:** 2014-01-02

**Authors:** Chikako Shinga-Ishihara, Yukie Nakai, Peter Milgrom, Kaori Murakami, Michiyo Matsumoto-Nakano

**Affiliations:** 1 Department of Pediatric Dentistry, Okayama University Graduate School of Medicine, Dentistry and Pharmaceutical Sciences, Okayama, Japan; 2 Department of Oral Health Sciences, University of Washington, Seattle, WA, USA

**Keywords:** Food frequency questionnaire, Cariogenic food, Diet, Reliability, Validity, Mutans streptococci

## Abstract

**Background:**

Diet is a major modifiable contributing factor in the etiology of dental caries. The purpose of this paper is to examine the reliability and cross-cultural validity of the Japanese version of the Food Frequency Questionnaire to assess dietary intake in relation to dental caries risk in Japanese.

**Methods:**

The 38-item Food Frequency Questionnaire, in which Japanese food items were added to increase content validity, was translated into Japanese, and administered to two samples. The first sample comprised 355 pregnant women with mean age of 29.2 ± 4.2 years for the internal consistency and criterion validity analyses. Factor analysis (principal components with Varimax rotation) was used to determine dimensionality. The dietary cariogenicity score was calculated from the Food Frequency Questionnaire and used for the analyses. Salivary mutans streptococci level was used as a semi-quantitative assessment of dental caries risk and measured by Dentocult SM. Dentocult SM scores were compared with the dietary cariogenicity score computed from the Food Frequency Questionnaire to examine criterion validity, and assessed by Spearman’s correlation coefficient (r_s_) and Kruskal-Wallis test. Test-retest reliability of the Food Frequency Questionnaire was assessed with a second sample of 25 adults with mean age of 34.0 ± 3.0 years by using the intraclass correlation coefficient analysis.

**Results:**

The Japanese language version of the Food Frequency Questionnaire showed high test-retest reliability (ICC = 0.70) and good criterion validity assessed by relationship with salivary mutans streptococci levels (r_s_ = 0.22; p < 0.001). Factor analysis revealed four subscales that construct the questionnaire (solid sugars, solid and starchy sugars, liquid and semisolid sugars, sticky and slowly dissolving sugars). Internal consistency were low to acceptable (Cronbach’s alpha = 0.67 for the total scale, 0.46-0.61 for each subscale). Mean dietary cariogenicity scores were 50.8 ± 19.5 in the first sample, 47.4 ± 14.1, and 40.6 ± 11.3 for the first and second administrations in the second sample. The distribution of Dentocult SM score was 6.8% (score = 0), 34.4% (score = 1), 39.4% (score = 2), and 19.4% (score = 3). Participants with higher scores were more likely to have higher dietary cariogenicity scores (p < 0.001; Kruskal-Wallis test).

**Conclusions:**

These results provide the preliminary evidence for the reliability and validity of the Japanese language Food Frequency Questionnaire.

## Background

Diet is a major modifiable contributing factor in the etiology of dental caries. In a healthy individual, new carious lesions do not develop and active caries lesions arrest with lower consumption of cariogenic foods. The frequent consumption of fermentable carbohydrate, especially sugar, has an important role in development of dental caries [1-3]. The Vipeholm Study, in which hospitalized mentally ill patients in Sweden were fed large amounts of sticky sugar sweetened foods, demonstrated the primary role of diet in the pathogenesis of dental caries [3]. Palmer and colleagues, using a short diet survey focused on the frequency of sweetened food intake, that children whose teeth were heavily colonized by the dental caries pathogen *Streptococcus mutans* had higher food cariogenicity scores [4]. Recently, Evans and colleagues reported preliminary results on a diet questionnaire including sugar sweetened beverages and focused younger children in an attempt to discriminate between children with and without severe early childhood caries [5]. A snacking culture, in which sweetened foods and beverages are consumed frequently, has increasingly been adopted in Asian countries as economic growth increased access to foreign culture.

Previous research examining the relationship between diet/nutrition and oral health among the Japanese used questionnaires that were not specifically designed for oral health studies. A semi-quantitative food frequency questionnaire [6], which consists of 5 food categories such as main meals, sugar, main dish, salt, and oil intake consumed during preceding week, was used to examine the relationship between the intake of dairy products and root caries in the elderly [7]. No assessments of reliability and limited data on validity were reported. A diet history questionnaire, consisting of 110 food items selected mainly from a food list used in National Nutrition Survey of Japan, was developed for use in health education. Tanaka and colleagues used this diet history questionnaire to hypothesize a negative relationship between tooth loss prevalence and the intake of magnesium [8], and also between tooth loss and the insoluble fiber foods [9] among Japanese women. However, the reliability of this instrument was not reported. Validity was established by comparison with 3-day diet record [10]. The Mini Nutrition Assessment (MNA) short-form [11,12] was used to assert the relationship between oral health status, swallowing function, nutritional status, cognitive ability and the activities of daily living [13]. The MNA is a well-known nutritional screening instrument designed for older people and the reliability and cross-cultural validity has been tested in Europe and USA [11]. Its reliability and validity has been partially assessed [14].

The interest in dietary counseling or interventions to reduce caries risk has been rising on a dental practice basis in Japan, although such approaches are not commonly done yet [15]. Simple and dietary instruments specifically designed to be used in epidemiological or interventional studies in Japan are needed.

The Food Frequency Questionnaire was designed specifically for dental studies and assesses the frequency of snacking and cariogenic quality of snacks [16]. It has demonstrated reliability and validity.

The purpose of this paper is to present preliminary evidence of reliability and cross-cultural validity of a culturally appropriate Japanese version of the English language Food Frequency Questionnaire [16]. The specific objectives were: (1) to investigate its construct validity through a factor analysis and examination of internal consistency; (2) to determine test-retest reliability; and (3) to assess criterion validity in terms of the relationship with salivary mutans streptococci levels.

## Methods

### Participants

As part of a larger study of mother-child transmission of mutans streptococci, pregnant women at the time of enrollment were recruited at the Miyake Obstetrics and Gynecology Clinic in Okayama prefecture (western Japan) between January 2004 and June 2005. To be included, the participants had to be able to communicate in Japanese and have visited the OB-GYN clinic at least once during the third to fifth month of pregnancy. Participants were excluded if they reported antibiotic use during the prior month. The subjects comprised 355 pregnant women aged 19–43 years (mean 29.2 ± 4.2 years). A separate convenience sample of 25 women aged 28–41 years (mean 34.0 ± 3.0 years) was recruited to participate in the test-retest reliability analysis via Ishihara Dental Clinic in Okayama based on the following criteria: the same age range between 19–43 years as the population for the validity analysis, absence of any disease that may influence nutritional status, the ability to complete the questionnaire.

The Okayama University Institutional Review Board approved the study and all participants gave their informed consent.

### Measures

#### Food frequency questionnaire

**Figure 1:**
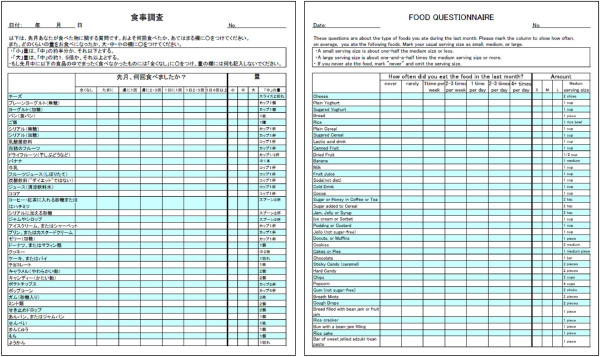
Japanese version of the food frequency questionnaire and its English translation.

The original English language questionnaire consists of 35 items requiring participants to recall how often, on average, they have consumed a given caries-related food during the past month (7 points, 0–6, categories ranging from “never” to “4 or more times per day”), and has established reliability and validity [16]. The English language questionnaire was translated in Japanese by a native speaker and then back translated into English by another native speaker to ensure comparability to the original form. Of them, 2 items, applesauce and breakfast drinks, were excluded because they were not familiar to the Japanese. Five items of traditional fresh and semi-dry confectioneries commonly consumed in Japan, such as bread filled with bean jam or fruit jam (*An-pan* or *Jam-pan*), rice cracker (*Senbei*), bun with a bean-jam filling (*Manju*), rice cake (*Mochi*), and bar of sweet jellied adzuki-bean paste(*Youkan*), were added to increase content validity. The Japanese version with 38 food items was then pretested and the translation modified. The final questionnaire in Japanese is in Figure 1. The questionnaire was scored by multiplying the frequency score for each item by a cariogenicity rating (3 points, 0–2, “possibly cariostatic” to “highly cariogenic”) based on the Palmer cariogenicity classification [17] and then summing to create the overall score that can possibly range from 0 to 378.

#### Salivary mutans streptococci levels

Salivary mutans streptococci level was assessed using the Dentocult SM Strip Mutans (Orion Diagnostica, Espoo, Finland). Participants refrained from having eating or drinking or brushing their teeth for at least 1 h prior to sampling. Each chewed a piece of paraffin for 1 minute and then swallowed the secreted saliva. Then the roughened side of the strip designed for salivary mutans assay was gently pressed against the tongue. The inoculated salivary strip was inserted into the culturing vial of Dentocult SM. Vials were transported to the lab for incubation. After incubation at 37°C for 48 h, bacterial growth on the strip was assessed in comparison with the density chart provided by the manufacturer by a single researcher (C.S.). The intra-rater reliability of the assessments were established as follows; 20 strips of Dentocult SM which had been assessed were re-assessed a week later by the same researcher blinded to the previous result, and then the agreement between those results was calculated prior to study implementation. The resulting reliability was high (κ > 0.8). According to the manufacturer, categories of Strip Mutans were ‘0-1′, ‘2′, ‘3′, correspond to <10^5^, 10^5^-10^6^, and >10^6^ CFU/mL of saliva, respectively.

### Procedures

For the validity study, the participants in the larger interventional study [18] completed the Food Frequency Questionnaire and had their saliva sampled during an initial visit. In the reliability study, the participants completed the questionnaire in the waiting room. They were given a second copy of the questionnaire and asked to complete it again one week later and bring or mail back. This interval was chosen because the questions focused on the diet at a particular time and recall would obviously be worse over time.

### Analysis

Distributions of the 38 items were examined to assess the degree of missing data. Although a total 400 participants were recruited, 355 questionnaires were complete and used in the validity sample. No missing data was found in test-retest reliability sample. Data were entered into the computer and checked for accuracy. Data management and analyses were conducted using SPSS ver. 19 (IBM SPSS Inc.). The internal consistency and test-retest reliability of the Japanese language Food Frequency Questionnaire were assessed using Cronbach’s alpha and intraclass correlation coefficient (ICC), respectively. Newly developed measures can be accepted with Cronbach’s alpha of >0.5, otherwise 0.7 should be the threshold [19]. ICCs were interpreted using the following criteria: ICC < 0.4, poor, 0.4 < ICC < 0.75, fair or good, ICC > 0.75, excellent [20]. Factor analysis was used to examine dimensionality. Principal components analysis with Varimax rotation was employed [21]. Kaiser’s criterion (eigenvalues > 1.0) and a visual examination of the scree plot were utilized to determine the number of components to retain. Internal consistency was assessed for the full scale and subscales developed from the factor analysis. Criterion validity was assessed by examining the relationship between the dietary cariogenicity score and Dentocult SM category using Spearman’s correlation coefficient (r_s_) and the Kruskal-Wallis test.

## Results

Finally 355 pregnant women completed the questionnaire. Forty-five women were excluded from the validity study because the questionnaires were not complete. The distribution of frequency score in each food item is given in Table 1. The questionnaire took about 3–5 minutes to complete.

**Table 1:** Distribution of frequency scores in each food item (n = 355)

**Food item**	**% distribution of frequency scores**						
	**0 (never)**	**1 (rarely)**	**2 (1/wk)**	**3 (2-3/wk)**	**4 (1/dy)**	**5 (2-3/dy)**	**6 (4+/dy)**
*Solid sugars*							
Donuts, or muffinns	52.7	40.3	3.9	2.5	0.6	0	0
Pudding or custard	39.0	49.4	6.8	4.5	0.3	0	0
Bread filled with bean	63.3	30.8	2.3	3.4	0.3	0	0
jam or fruit jam							
Bar of sweet jellied	90.7	7.9	1.1	0.3	0	0	0
adzuki-bean paste							
*Solid and starchy sugars*							
Rice cake	74.9	20.8	2.8	1.1	0.3	0	0
Bun with a bean-jam filling	48.2	45.1	4.8	2.0	0	0	0
Popcorn	89.0	10.5	0.3	0.3	0	0	0
Rice cracker	40.0	44.8	7.0	7.0	0.8	0.3	0
Chocolate	31.1	46.9	8.5	11.9	1.7	0	0
*Liquid and semisolid sugars*							
Cold drink	27.7	41.5	5.9	15.0	7.6	1.7	0.6
Soda (not diet)	43.2	39.5	5.4	8.5	2.3	0.8	0.3
Ice cream or sorbet	15.5	48.2	13.8	18.3	3.9	0.3	0
*Sticky and slowly dissolving sugars*							
Hard candy	37.7	41.1	2.0	10.1	5.1	3.1	0.8
Gum (not sugar-free)	64.8	23.1	4.2	3.7	1.4	1.7	1.1
Sugar or honey in coffee/tea	49.4	19.9	4.3	10.5	13.9	1.4	0.6
Sticky candy (caramel)	82.0	15.2	1.1	1.1	0.3	0.3	0
*Other*							
Cheese	24.4	45.9	10.5	15.6	2.8	0.6	0.3
Plain yoghurt	56.4	17.3	5.4	11.9	8.5	0.3	0.3
Sugared yoghurt	27.9	29.9	11.7	20.2	9.4	0.9	0
Bread	4.3	15.1	8.2	30.4	36.9	2.8	2.3
Rice	1.2	2.7	0.6	4.4	17.7	69.3	4.1
Plain cereal	95.2	3.4	0.9	0.6	0	0	0
Sugared cereal	86.9	10.2	1.1	0.9	0.9	0	0
Lactic acid drink	37.5	35.2	8.5	10.5	7.4	0.3	0.6
Canned fruit	68.5	23.9	4.0	3.1	0.6	0	0
Dried fruit	76.6	14.1	2.0	2.8	3.7	0.6	0.3
Banana	26.3	42.9	8.5	16.1	4.5	0.6	1.1
Milk	11.6	11.9	3.4	22.0	36.2	13.0	2.0
Fruit juice	51.0	28.6	5.4	8.2	4.5	1.7	0.6
Cocoa	64.9	19.8	4.5	5.1	5.4	0.3	0
Sugar added to cereal	98.9	1.1	0	0	0	0	0
Jam, jelly or syrup	57.6	22.3	4.2	9.6	5.6	0.3	0.3
Jello (not sugar-free)	60.6	30.0	3.0	5.1	0.6	0.6	0
Cookies	43.6	43.9	6.2	4.8	1.4	0	0
Cakes or pies	27.6	65.4	5.4	1.7	0	0	0
Chips	38.0	50.1	8.2	3.4	0.3	0	0
Breath mints	82.3	12.4	1.7	1.7	1.7	0.3	0
Cough drops	94.9	2.8	0.6	0.6	0	0.8	0.3

Due to rounding error, some totals are not 100%.

### Construct validity and internal consistency

As results of the factor analysis for the pregnant women sample, by Kaiser’s criterion, 13 factors had eigenvalues > 1, which together accounted for 56.8% of variance in total scores. The scree plot confirmed the retention of the first four factors. Table 2 shows the factor loadings after rotation and the four factors. Factor I was termed *solid sugars subscale* because it included items pertaining to: donuts/muffins, pudding/custard, bread filled with bean jam or fruit jam, and bar of sweet jellied adzuki-bean paste. Factor II was termed *solid and starchy sugars subscale* because it included items pertaining to: rice cake, bun with a bean-jam filling, popcorn, rice cracker, and chocolate. Factor III was termed *liquid and semisolid sugars subscale* including items such as cold drink, not-diet soda, and ice cream/sorbet. Factor IV was termed *sticky and slowly dissolving sugars subscale* including such as hard candy, not-sugar-free gum, sugar/honey in coffee/tea, and sticky candy/caramel. Cronbach’s alpha for the total scale was 0.67. For each subscale, alphas were 0.61, 0.58, 0.56, and 0.46, respectively. For each subscale, mean cariogenicity subscores were 3.9 (SD = 3.6, range 0–24), 5.5 (SD = 4.1, range 0–26), 7.6 (SD = 5.2, range 0–30), and 6.6 (SD = 6.1, range 0–36), respectively.

**Table 2:** Factor loadings, eigenvalues, variance explained from principal component analysis with Varimax rotation, and Cronbach’s alpha for each factor for the Japanese version of the food frequency questionnaire in Japanese (n = 355)

	**Factor loading component**			
	**I**^**a**^	**II**^**b**^	**III**^**c**^	**IV**^**d**^
Donuts, or muffins	**0.704**	0.030	0.111	0.091
Pudding or custard	**0.624**	-0.111	0.322	0.134
Bread filled with bean jam or fruit jam	**0.622**	0.712	0.095	-0.229
Bar of sweet jellied adzuki-bean paste	**0.480**	0.286	-0.046	0.025
Rice cake	-0.109	**0.705**	-0.111	-0.106
Bun with a bean-jam filling	0.379	**0.588**	-0.022	0.013
Popcorn	0.095	**0.554**	0.085	0.184
Rice cracker	0.238	**0.472**	0.033	0.063
Chocolate	0.173	**0.420**	0.124	0.182
Cold drink	0.075	0.019	**0.696**	0.129
Soda (not diet)	0.148	-0.011	**0.614**	0.133
Ice cream or sorbet	0.183	0.038	**0.612**	0.011
Hard candy	-0.016	0.043	0.176	**0.679**
Gum (not sugar-free)	0.083	-0.026	0.221	**0.648**
Sugar or honey in coffee or tea	-0.112	0.065	0.083	**0.512**
Sticky candy (caramel)	0.071	0.157	-0.105	**0.415**
Cheese	0.139	-0.009	-0.053	0.007
Plain yoghurt	-0.052	0.078	-0.094	0.037
Sugared yoghurt	0.208	-0.076	-0.134	0.127
Bread	0.192	0.047	-0.036	0.020
Rice	-0.050	0.039	0.006	-0.039
Plain cereal	-0.073	0.076	0.048	-0.051
Sugared cereal	0.033	-0.047	0.140	-0.007
Lactic acid drink	0.004	0.103	0.042	0.071
Canned fruit	0.072	0.037	0.042	0.052
Dried fruit	0.123	-0.112	-0.085	0.088
Banana	0.165	-0.113	-0.213	-0.012
Milk	-0.045	-0.065	-0.101	0.067
Fruit juice	-0.046	-0.082	0.155	0.140
Cocoa	-0.023	0.177	-0.208	0.347
Sugar added to cereal	-0.048	0.067	0.070	-0.011
Jam, jelly or syrup	-0.184	0.337	0.244	-0.100
Jello (not sugar-free)	0.180	-0.018	0.326	-0.081
Cookies	0.341	0.269	-0.026	0.140
Cakes or pies	0.382	0.235	0.158	0.171
Chips	0.072	0.320	0.174	0.069
Breath mints	0.155	0.071	-0.069	0.347
Cough drops	-0.096	0.019	0.064	0.042
Eigenvalue	4.055	2.183	1.898	1.827
% variance	10.671	5.745	4.996	4.807
Cronbach’s alpha	0.61	0.58	0.56	0.46

^a^Factor I is called “*solid sugars subscale*”.

^b^Factor II is called “*solid and starchy sugars subscale*”.

^c^Factor III is called “*liquid and semisolid sugars subscale*”.

^d^Factor IV is called “*sticky and slowly dissolving sugars subscale*”.

Factor loadings over 0.400 appear in bold.

### Test-retest reliability

**Figure 2:**
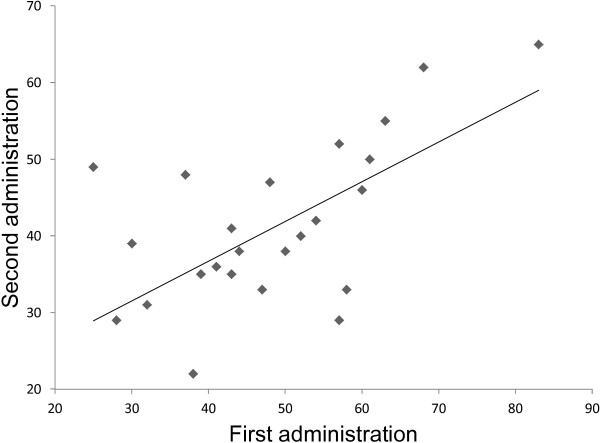
**Scatter plot and correlation relationship of dietary cariogenicity score between the first and second administrations for test-retest reliability.** ICC = 0.70, n = 25.

The mean dietary cariogenicity scores derived from the Food Frequency Questionnaire in Japanese for the first and second administrations were 47.4 ± 14.1 (range 25–83) and 40.6 ± 11.3 (range 19–65). The test-retest reliability was high (ICC = 0.70). Figure 2 is a scatter plot of the relationship between those scores.

### Criterion validity

**Figure 3:**
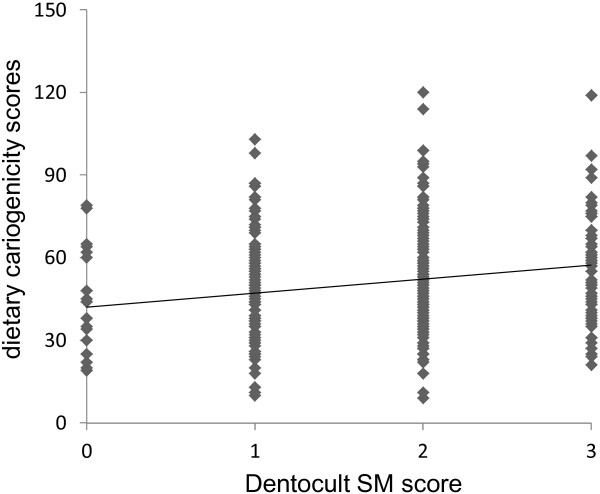
**Scatter plot and correlation relationship between dietary cariogenicity and Dentocult SM scores.** r_s_ = 0.22, n = 355, p < 0.001.

The mean dietary cariogenicity score derived from the Japanese version of the Food Frequency Questionnaire was 50.8 ± 19.5 (range 9–120) in the pregnant women sample. The distribution of Dentocult SM scores was 6.8% (n = 24, score = 0), 34.4% (n = 122, score = 1), 39.4% (n = 140, score = 2), and 19.4% (n = 69, score = 3). Figure 3 shows a scatter plot of the relationship between dietary cariogenicity and Dentocult SM scores. The dietary cariogenicity score was positively correlated with measure of salivary mutans streptococci (r_s_ = 0.22, p < 0.001). The cariogenicity subscore was also positively correlated with measure of salivary mutans streptococci in each subscale (r_s_ = 0.17, p < 0.01 for *solid sugars subscale,* r_s_ = 0.192, p < 0.001 for *solid and starchy sugars subscale,* r_s_ = 0.169, p < 0.01 for *liquid and semisolid sugars subscale,* r_s_ = 0.086, p = 0.106 for *sticky and slowly dissolving sugars subscale*). Table 3 gives the mean dietary cariogenicity score for each category of Dentocult SM score. Individuals with higher scores were more likely to have higher dietary cariogenicity scores (p < 0.001; Kruskal-Wallis test).

**Table 3:** Mean dietary cariogenicity score* for each category of Dentocult SM scores (n = 355)

	**Dentocult SM score**			
	**0**	**1**	**2**	**3**
Mean ± SD	41.0 ± 18.0	47.5 ± 18.3	51.8 ± 19.5	57.8 ± 20.1
n	24	122	140	69

P < 0.001, Kruskal-Wallis test.

*The dietary cariogenicity score was calculated by multiplying the frequency score for each item by a cariogenicity rating (3 points, 0–2, “possibly cariostatic” to “highly cariogenic”) based on the Palmer cariogenicity classification and then summing to create the overall score.

## Discussion

This study provides preliminary evidence for reliability and cross-cultural validity of the Japanese language version of the Food Frequency Questionnaire specifically created for studies of dental caries. Factor analysis suggested four subscales: “*solid sugars*”, “*solid and starchy sugars*”, “*liquid and semisolid sugars*”, and “*sticky and slowly dissolving sugars*”, corresponding to an established classification of sugary foods by extent of oral retention [17]. The reliability and validity of the Japanese language Food Frequency Questionnaire is good, as compared with other food frequency questionnaires [22]. Criterion validity was established for the Japanese language version by examining the association between the Food Frequency Questionnaire cariogenicity score and salivary mutans streptococci levels. The Dentocult SM measure has been shown to be a reasonable surrogate for dental caries [23,24]. The questionnaire is simple and is quickly completed. The questionnaire containing food items, most of which had derived from USA, reflected caries risk indicator among Japanese. The finding implies that this questionnaire has potential to compare the caries risk levels among the other cultures where these foods have become part of the diet.

The validation study was carried out as part of a larger study of the transmission of mutans streptococci from mother to child [18]. This transmission is an important factor in the development of tooth decay in children. Before any intervention in this study, the women completed the questionnaire. Dietary preferences often change in pregnant women and may lead to frequent intake of cariogenic foods. Thus, they were chosen for this initial validation study.

This study has strengths. An extensively studied instrument designed specifically for looking at the relationship between diet and tooth decay served as the basis for the Japanese instrument. Moreover, the microbiological assessment was used as a surrogate gold standard for dental caries. It is a well-established measure of the risk of transmission from mother to child [18,25]. However, other factors such as hormonal changes and genetic disposition may have moderated the results. Future work on the instrumentation in Japanese should include a broader group of participants. Nevertheless, the Japanese version of the Food Frequency Questionnaire exhibited good test-retest reliability, acceptable internal consistency, and good construct and criterion validity. These preliminary findings suggest the applicability of the Japanese version of the instrument in dental caries research.

## Conclusions

The Japanese version of the Food Frequency Questionnaire is a reliable and valid instrument to assess dietary intake in relation to dental caries risk, and can operate the same in Japan as it does in USA culture.

## Competing interests

The authors declare that they have no competing interests.

## Authors’ contributions

CS contributed to data collection, statistical analyses, and writing of the manuscript. YN was principal investigator of the research, conceptualized the paper, conducted statistical analyses with primary responsibility, and wrote the final paper. PM supervised preparing the conceptual framework for the paper and contributed to the overall manuscript. KM contributed to data collection and statistical analyses. MMN oversaw the procedures. All authors read and approved the final manuscript.

## Pre-publication history

The pre-publication history for this paper can be accessed here:

http://www.biomedcentral.com/1472-6831/14/1/prepub
